# Occupational Noise Exposure and Age Correction: The Problem of Selection Bias

**DOI:** 10.3390/ijerph6123023

**Published:** 2009-12-01

**Authors:** Robert A. Dobie

**Affiliations:** 1 Department of Otolaryngology, Head and Neck Surgery, University of Texas, Health Science Center at San Antonio, 7703 Floyd Curl Dr., San Antonio, TX 78229, USA; E-Mail: dobie@uthscsa.edu; Tel.: +1 (210) 567-5655; 2 Department of Otolaryngology, Head and Neck Surgery, University of California, Davis Medical Center, 2521 Stockton Blvd, Sacramento, CA 95818, USA

**Keywords:** age correction, noise-induced, age-related, selection bias, NIPTS

## Abstract

Selection bias often invalidates conclusions about populations based on clinical convenience samples. A recent paper in this journal [1] makes two surprising assertions about noise-induced permanent threshold shift (NIPTS): first, that there is more NIPTS at 2 kHz than at higher frequencies; second, that NIPTS declines with advancing age. Neither assertion can be supported with the data presented, which were obtained from a clinical sample; both are consistent with the hypothesis that people who choose to attend an audiology clinic have worse hearing, especially at 2 kHz, than people of the same age and gender who choose not to attend.

In a recent paper, Krishnamurti [1] analyzed the audiograms of 68 middle-aged and elderly patients attending an audiology clinic who had symmetrical sensorineural hearing loss and “significant histories of occupational noise exposure.” The audiograms were age-corrected by subtracting, at every frequency, the mean thresholds for people of the same age group and sex from a population-based survey. The corrected thresholds were considered by Krishnamurti to represent noise-induced permanent threshold shift (NIPTS). Analysis of variance revealed that NIPTS varied by both age and audiometric frequency: the largest NIPTS values were seen at younger ages and at 2 kHz.

Unfortunately, the age-corrected thresholds analyzed in this paper cannot be considered to represent noise-induced changes for two reasons. First, the criteria for “significant history of occupational noise exposure” were unstated (many “noisy” jobs do not reach the level of hazardous noise exposure). The second and more important problem is selection bias: the subjects were almost certainly self-selected for severity of hearing loss. Most people who choose to attend an audiology clinic believe that they have difficulty hearing, and most of them indeed are found to have substantial hearing impairment. Obviously, such patients will have hearing thresholds that are worse than those in the general population. Age-corrected thresholds will therefore represent the degree to which people who think they are hearing-impaired hear worse than people of the same age and sex in the general population. Such age-corrected thresholds would be positive (on the average), even for patients who had never had occupational noise exposure (higher thresholds mean worse hearing).

Middle-aged people hear much better than elderly people; mean audiometric thresholds increase with age. If the degree of hearing impairment that motivates attendance at an audiology clinic were constant across the age spectrum, it would follow that the age-corrected thresholds of elderly patients would be lower than the age-corrected thresholds of middle-aged patients. That is what we would expect whether or not the patients were noise-exposed, and that is what was found [1].

Audiometric thresholds are conventionally obtained at octave intervals from 0.25 kHz to 8 kHz, but these frequencies are not equally important for everyday communication. “Frequency-importance” functions routinely show that frequencies in the geometric center of the audiometric spectrum (1 to 2 kHz) are much more important than higher or lower frequencies, and that the single most important audiometric frequency is 2 kHz [2]. It is therefore no surprise that this is the frequency at which patients attending an audiology clinic differ most from people of the same age and sex in the general population; when there is substantial hearing loss at 2 kHz, people begin to have real problems in everyday life. That is what we would expect whether or not the patients were noise-exposed, and that is what was found [1]. Because of selection bias, Krishnamutri’s conclusions about variations of NIPTS with age and audiometric frequency cannot be considered as valid.
